# *mPeriod2*^*Brdm1*^ and other single *Period* mutant mice have normal food anticipatory activity

**DOI:** 10.1038/s41598-017-15332-6

**Published:** 2017-11-14

**Authors:** Julie S. Pendergast, Robert H. Wendroth, Rio C. Stenner, Charles D. Keil, Shin Yamazaki

**Affiliations:** 1 Department of Biology, University of Kentucky, Lexington, Kentucky USA; 2 Department of Biological Sciences, Vanderbilt University, Nashville, Tennessee USA; 3 Present Address: Department of Neuroscience, University of Texas Southwestern, Dallas, TX USA

## Abstract

Animals anticipate the timing of food availability via the food-entrainable oscillator (FEO). The anatomical location and timekeeping mechanism of the FEO are unknown. Several studies showed the circadian gene, *Period 2*, is critical for FEO timekeeping. However, other studies concluded that canonical circadian genes are not essential for FEO timekeeping. In this study, we re-examined the effects of the *Per2* ^*Brdm1*^ mutation on food entrainment using methods that have revealed robust food anticipatory activity in other mutant lines. We examined food anticipatory activity, which is the output of the FEO, in single *Period* mutant mice. Single *Per1*, *Per2*, and *Per3* mutant mice had robust food anticipatory activity during restricted feeding. In addition, we found that two different lines of *Per2* mutant mice (*ldc* and *Brdm1*) anticipated restricted food availability. To determine if FEO timekeeping persisted in the absence of the food cue, we assessed activity during fasting. Food anticipatory (wheel-running) activity in all *Period* mutant mice was also robust during food deprivation. Together, our studies demonstrate that the *Period* genes are not necessary for the expression of food anticipatory activity.

## Introduction

The food-entrainable oscillator (FEO) is an enigmatic circadian pacemaker that is entrained by temporally restricted food availability^1^. During daytime restricted feeding, mice display anticipatory activity (the output of the FEO) prior to food availability. The self-sustained nature of the FEO is evidenced by the persistence of anticipatory activity during fasting subsequent to restricted feeding. Numerous attempts to identify the locus of the FEO have been unsuccessful^2^. However, the FEO does not reside in the master circadian pacemaker in the suprachiastmatic nucleus (SCN) since food anticipatory activity persists in SCN-lesioned animals^3–5^.

Recent studies have shown that the molecular timekeeping mechanism of the FEO operates differently compared to canonical circadian oscillators (e.g. SCN, liver, lung). This was first demonstrated in homozygous *Clock* Δ19 mutant mice that have arrhythmic SCN-controlled nocturnal activity, but normal FEO-controlled food anticipatory activity^6^. Similarly, mice lacking both functional *Cryptochrome (Cry)1* and *Cry2*, or both *Period (Per)1* and *Per2*, or *Per1*, *Per2*, and *Per3* exhibit food anticipatory activity (albeit sometimes abnormal or with a non-24h period) when nocturnal activity is arrhythmic^7–9^.

However, several studies suggested that some canonical circadian genes are necessary for FEO timekeeping (Table 1). Three studies showed that food anticipatory activity was absent in *Per2* mutant mice (the *Brdm1* strain)^10–12^. Another study used mice with a conditional *Per2* allele and showed that total-body and liver-specific *Per2* mutant mice did not express FAA^13^. In contrast, Storch and Weitz showed that a different line of *Per2* mutant mice (the *ldc* strain) had robust food anticipatory activity^8^. In this study, we sought to re-examine the roles of the *Period* genes in food entrainment.

**Table 1:** Summary of previous studies of food anticipatory activity in *Period* mutant mice.

Genotype	Genetic background	Age (weeks)	Length of food availability	RF phase	Results	Reference
***Per1*** ^−/−^	C57BL/6 × 129SvEvBrd	12 to 28	8 h	ZT4–12	FAA present	10
	C57BL/6 × 129SvEvBrd	Not reported	6 h	ZT6–12	FAA present	11
	C57BL/6 J (>N12)	8	4 h	ZT4–8	FAA present	12
***mPer2*** ^***Brdm1***−/−^	C57BL/6 × 129SvEvBrd	12 to 28	8 h	ZT4–12	FAA absent	10
	C57BL/6 × 129SvEvBrd	Not reported	6 h	ZT6–12	FAA absent	11
	C57BL/6 J (>N12)	8	4 h	ZT4–8	FAA very weak or absent	12
***mPer2*** ^***ldc***−/−^	129/C57BL/6	7 to 9	3 h	ZT6–9	FAA present	8
***mPer1*** ^***ldc***−/−^ ***/ mPer2*** ^***ldc***−/−^	129	7 to 9	3 h	ZT6–9	FAA present	8
***mPer1*** ^***ldc***−/−^ ***/ mPer2*** ^***ldc***−/−^ ***/ mPer3*** ^−/−^	C57BL/6 J	9 to 20	6 h	ZT8–14	FAA present	9

^*^All studies were performed in 12 L:12D, except the triple *Per1/2/3* mutant mice study that was performed in 18L:6D. Wheel-running food anticipatory activity (FAA) was measured during restricted feeding (RF) in mice with intact (not lesioned) SCN. Results from studies where caloric restriction was combined with restricted feeding are not reported.

## Results

### *Period* mutant (ldc strain) mice have robust food anticipatory activity during restricted feeding and fasting

**Figure 1:**
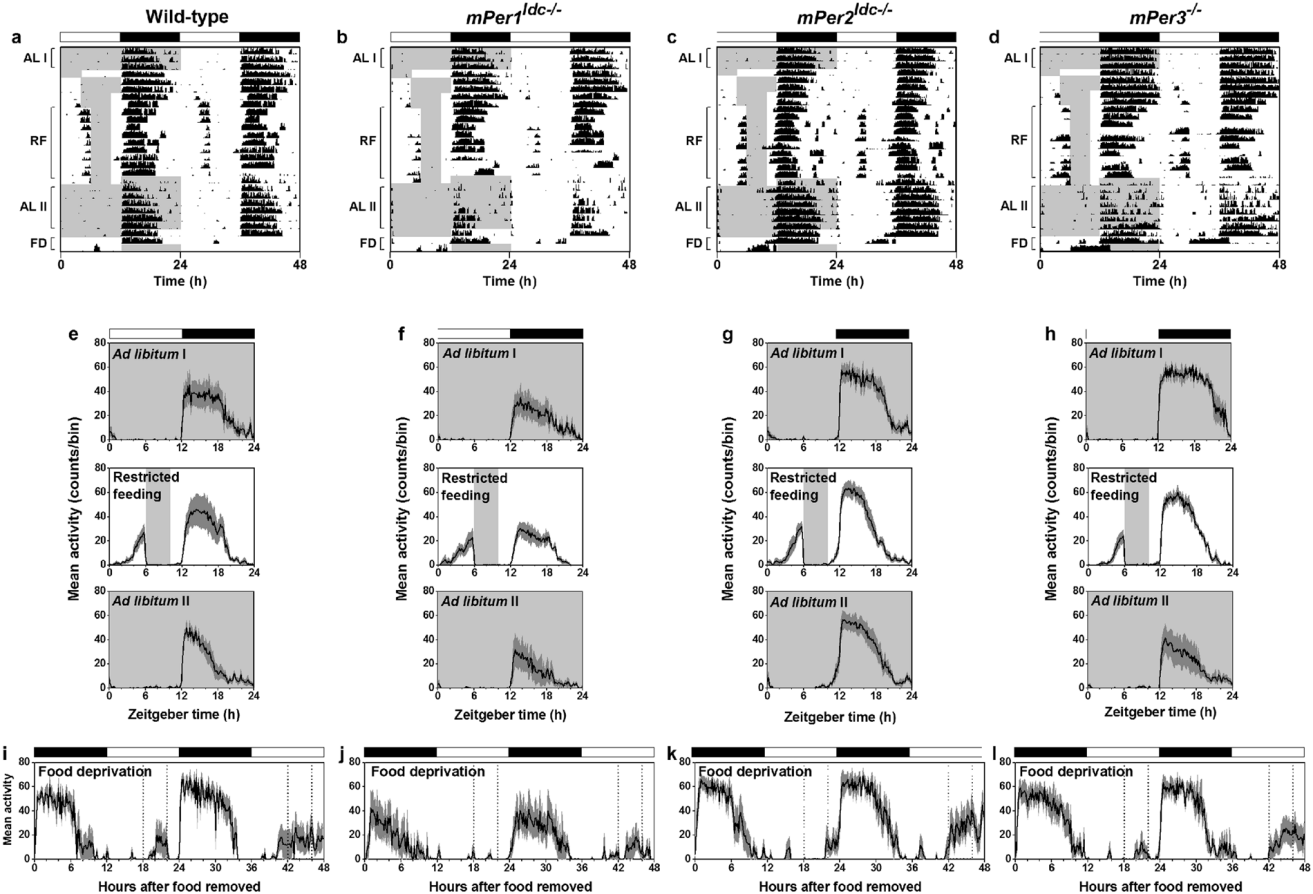
PERIOD-deficient (*ldc*) mice have robust wheel-running food anticipatory activity during restricted feeding and food deprivation. Representative double-plotted actograms (**a**–**d**) and group average activity profiles (**e**–**l**) of wild-type (**a**,**e**,**i**), *mPer1* ^*ldc*−/−^ (**b**,**f**,**j**), *mPer2* ^*ldc*−/−^ (**c**,**g**,**k**), and *mPer3* ^−/−^ (**d**,**h**,**l**) mice. The time when food was available is indicated by gray shading on the left half of each actogram and in the activity profiles. The light-dark cycle is indicated by the white and black bars, respectively. The black traces in the group activity profiles represent the mean number of wheel revolutions (in counts/10-minute bin) relative to the light-dark cycle where 0 is lights on and 12 is lights off. The SEM is shown in dark gray shading in each activity profile. ALI, RF, ALII, and FD in a-d indicate the days used to generate the activity profiles *ad libitum* I, restricted feeding, *ad libitum* II (**e**–**h**) and food deprivation (**i**–**l**), respectively. Forty-eight hours of continuous food deprivation is shown (**i**–**l**) and the dotted lines indicate when food was available during the preceding restricted feeding.

**Figure 2:**
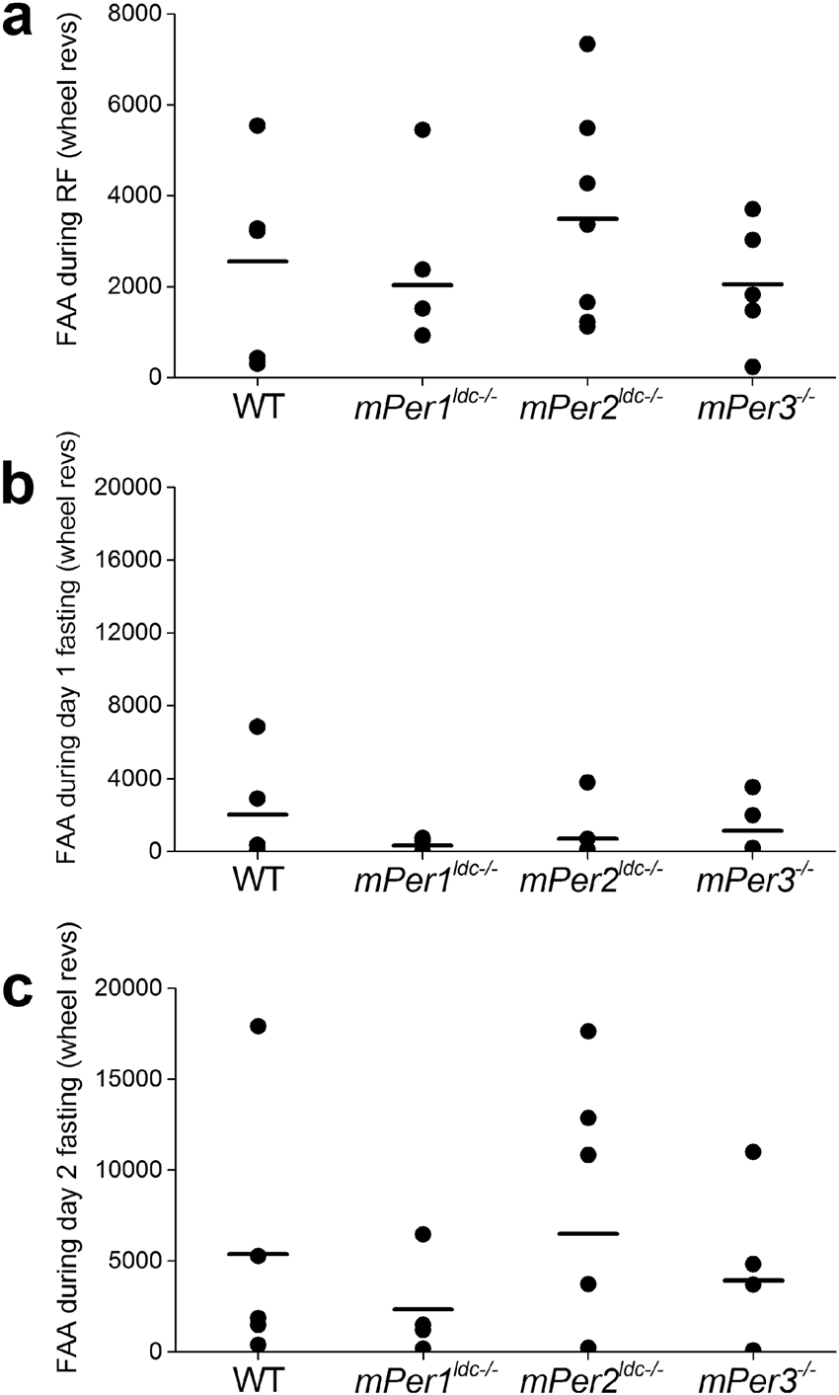
Food anticipatory activity from individual wild-type and *Period* mutant *(ldc strain*) mice. (**a**) Food anticipatory activity (FAA) during 9 days of restricted feeding (RF) of wild-type (n = 5), *mPer1* ^*ldc*−/−^ (n = 4), *mPer2* ^*ldc*−/−^ (n = 7), and *mPer3* ^−/−^ (n = 5) mice was determined by totaling the number of wheel revolutions per minute from 4 hours before feeding time to the end of feeding time (total of 8 hours). FAA during fasting was defined as the total number of wheel revolutions per minute from 4 hours before feeding time to the end of previous feeding time (total of 8 hours). Wheel-running FAA for each mouse was determined separately for the first (Day 1; **b**) or second (Day 2; **c**) day of fasting. Each black circle is data from one mouse. The mean of each group is a horizontal line.

We first determined if C57BL/6 J *Period1* (*mPer1* ^*ldc*−/−^), *Period2* (*mPer2* ^*ldc*−/−^), and *Period3* (*mPer3* ^−/−^) mutant mice^14^ expressed food anticipatory activity during daytime restricted feeding (ZT6-10) and subsequent fasting. During *ad libitum* feeding, all mice had minimal daytime wheel-running activity (Fig. 1: AL1; actograms of all mice shown in Figs. S1–S4). In contrast, during 4-h restricted feeding, wheel-running activity began 2 to 4 hours before feeding time and continued until food was provided at ZT6 in wild-type and *mPer1* ^*ldc*−/−^, *mPer2* ^*ldc*−/−^, and *mPer3* ^−/−^ mice (RF in Fig. 1, Fig. 2a). When mice were returned to *ad libitum* feeding, food anticipatory activity disappeared (Fig. 1: ALII). However, when we food deprived mice after 1 week of *ad libitum* feeding, food anticipatory activity reappeared at a similar phase in *Per* mutant and wild-type mice (Fig. 1: FD). As we previously reported, food anticipatory activity was weak or absent on the first day of fasting (Fig. 2b), but was robust in all genotypes on the second day of food deprivation (Fig. 2c).

To determine if genotype-specific differences in total daily activity affected the expression of food anticipatory activity, we expressed food anticipatory as a ratio of total daily activity (Fig. S5). We found that wild-type and *mPer1* ^*ldc*−/−^, *mPer2* ^*ldc*−/−^, and *mPer3* ^−/−^ mice had similar food anticipatory activity ratios during RF (Fig. S5a), day 1 fasting (Fig. S5b), and day 2 fasting (Fig. S5c). We also found that the ages of the mice were not correlated with their food anticipatory activity ratios (Fig. S6).

### *Period2* mutant (Brdm1 strain) mice have robust food anticipatory activity during restricted feeding and fasting

**Figure 3:**
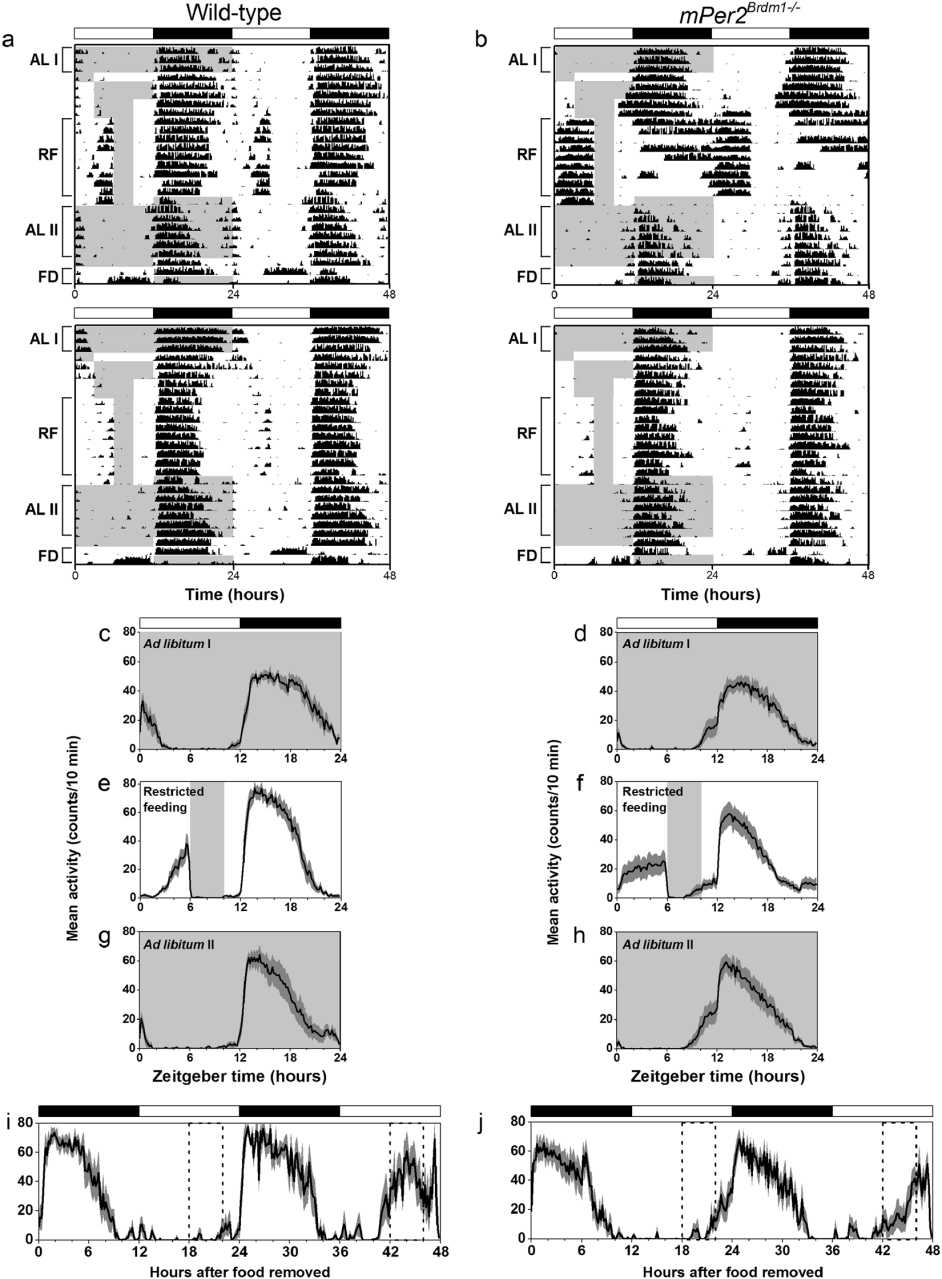
Food anticipatory activity in *Period2* mutant (*Brdm*) mice. Representative double-plotted actograms (**a**,**b**) and group average activity profiles (**c**–**j**) of wild-type (**a**,**c**,**e**,**g**,**i**) and *mPer2* ^*Brdm1*−/−^ (**b**,**d**,**f**,**h**,**j**) mice. The time when food was available is indicated by gray shading on the left half of each actogram and in the activity profiles. The light-dark cycle is indicated by the white and black bars, respectively. The black traces in the group activity profiles represent the mean number of wheel revolutions (in counts/10-minute bin) plotted relative to the light-dark cycle where 0 is lights on and 12 is lights off. The SEM is shown in dark gray shading in each activity profile. ALI, RF, ALII, and FD in a-d indicate the days used to generate the activity profiles *ad libitum* I (**c**,**d**), restricted feeding (**e**,**f**), *ad libitum* II (**g**,**h**) and food deprivation (**I**,**j**), respectively. Forty-eight hours of continuous food deprivation is shown (**I**,**j**) and the dotted lines indicate when food was available during the preceding restricted feeding.

**Figure 4:**
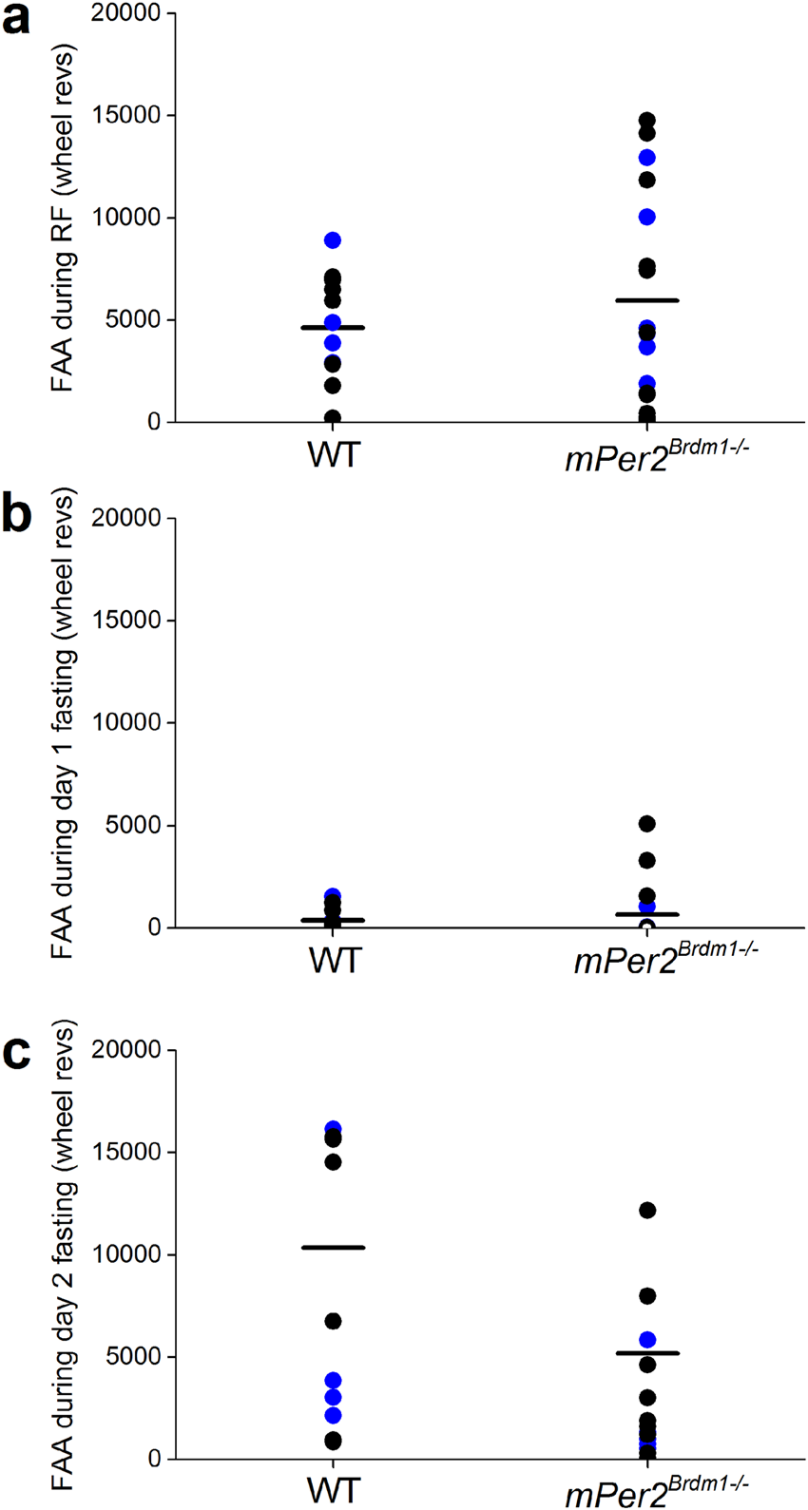
Food anticipatory activity from individual wild-type and *Period* mutant *(Brdm strain*) mice. (**a**) Food anticipatory activity (FAA) during 9 days of restricted feeding (RF) of wild-type (n = 12) and *mPer2* ^*Brdm1*−/−^ (n = 17) mice was determined by totaling the number of wheel revolutions per minute from 4 hours before feeding time to the end of feeding time (total of 8 hours). FAA during fasting was defined as the total number of wheel revolutions per minute from 4 hours before feeding time to the end of previous feeding time (total of 8 hours). Wheel-running FAA for each mouse was determined separately for the first (Day 1; **b**) or second (Day 2; **c**) day of fasting. Black circles are individual mice fed from ZT6-10. Blue circles are individual mice fed from ZT5-9. The mean of each group is a horizontal line.

Three previous studies found that food anticipatory activity was absent or very weak in *mPer2* ^*Brdm1*−/−^ mice^10–12^. The *mPer2* ^*Brdm1*−/−^ mice are a distinct strain from the *mPer2* ^*ldc*−/−^ mice we used in our first experiment^14,15^. These are 2 distinct lines of *Per2* mutants produced by different laboratories. *mPer2* ^*Brdm1*−/−^ mice express a mutant transcript that lacks most of the PAS domain, while *mPer2* ^*ldc*−/−^ mice are null mutants that do not express PERIOD2 protein^14,15^. Moreover, in 2 studies of food anticipatory activity in the *mPer2* ^*Brdm1*−/−^ strain, the mice were on a hybrid C57BL/6 × 129S5/SvEvBrd genetic background^10,11^. Thus, we next performed daytime restricted feeding in *mPer2* ^*Brdm1*−/−^ mice on a hybrid genetic background (Fig. 3; actograms of all mice shown in Figs S7–S13). During *ad libitum* feeding, wild-type and *mPer2* ^*Brdm1*−/−^ mice had minimal daytime activity (Fig. 3: ALI). During 4-h restricted feeding (ZT6-10; ZT5-9 in Figs S7–S9), the wheel-running activity of wild-type and *mPer2* ^*Brdm1*−/−^ mice increased prior to feeding (Fig. 3: RF; Fig. 4a). Food anticipatory activity disappeared during *ad libitum* feeding after restricted feeding (Fig. 3: ALII). Robust daytime activity reappeared at the predicted phase in some *mPer2* ^*Brdm1*−/−^ mice during the second, but not first, day of fasting (Fig. 3: FD; Fig. 4b, c). Later we released the mice into constant darkness and confirmed that *mPer2* ^*Brdm1*−/−^ mice had short free-running periods of activity compared to wild-type mice as previously reported (Figs S7–S13)^15^.

The food anticipatory activity ratio, which normalizes anticipatory activity to daily activity levels, was robust in wild-type and *mPer2* ^*Brdm1*−/−^ mice during restricted feeding (Fig. S14a) and day 2 of fasting (Fig. S14c). Moreover, food anticipatory activity occurred on day 2 of fasting only in mice that had been previously exposed to restricted feeding. Naïve (never exposed to restricted feeding) *mPer* ^*Brdm1*−/−^ mice did not have elevated daytime activity during 48-h fasting (Fig. S15). The ages of the mice were also not correlated with their food anticipatory activity ratios (Fig. S16).

## Discussion

The *Period* genes are critical for timekeeping in canonical circadian clocks. For example, mice with non-functional *Per1* and *Per2* have arrhythmic SCN and locomotor activity, while mice lacking functional *Per3* have altered circadian rhythms in peripheral tissue clocks^14,16^. The molecular timekeeping mechanism of the FEO, however, is still unknown. More than a decade ago, mice without functional *Per2* were reported to lack food anticipatory activity during restricted feeding^10^. This study (and 2 other studies that confirmed this finding in *mPer2* ^*Brdm1*−/−^ mice) strongly suggested that the FEO uses a canonical molecular timekeeping similar to the SCN and peripheral clocks^10–12^. However, other studies have shown that canonical circadian genes are not required for FEO timekeeping. For example, both double *Per1/2* and triple *Per1/2/3* mutant mice have robust anticipatory activity during restricted feeding. In this study, we re-examined these discrepant results and showed that single *Per1*, *Per2*, *and Per3* mutant mice have food anticipatory activity during restricted feeding. Moreover, we found that 2 different lines of *Per2* mutant mice anticipated restricted food availability.

Three previous studies did not observe food anticipatory activity during restricted feeding in *mPer2* ^*Brdm1*−/−^ mice. These results are in contrast with our study where we observed clear food anticipatory during restricted feeding in this same line of *Per2* mutant mice. We hypothesize that subtle differences in experimental conditions account for this discrepancy. First, we provided food for only 4-h per day, while previous studies performed 6-h or 8-h restricted feeding [but see Li *et al*. (2015) for 4-h restricted feeding]. Second, we fed the mice beginning at ZT5 or ZT6, while previous studies sometimes began restricted feeding at ZT4^10,12^. We previously showed that the phase of restricted feeding regulated the robustness of food anticipatory activity so incremental changes in the phase of restricted feeding could permit or conceal the expression of anticipatory activity^17^. Finally, we measured wheel-running food anticipatory activity, which enhances food anticipatory activity compared to mice without running wheels^17^. Thus, it appears that the combination of an aggressive restricted feeding protocol (4-h/day) at mid-day phases (ZT6-10 and ZT5-9) with running wheels permitted the detection of food anticipatory activity in *mPer2* ^*Brdm1*−/−^ mice in the current study.

Notably, we did observe individual differences in the robustness of food anticipatory activity during restricted feeding in both *mPer2* ^*Brdm1*−/−^ mice and their wild-type littermates. This could be due to the mixed genetic background of these mice. We have also previously shown that food anticipatory is more robust in long photoperiods (18 L:6D) compared to 12 L:12D. If we had performed our experiments in 18L:6D, we may have reduced the individual variability in the robustness of food anticipatory activity in both wild-type and *mPer2* ^*Brdm1*−/−^ mice^17,18^.

We also examined food anticipatory activity during fasting to determine if the output of the FEO was sustained in the absence of the temporal food cue. We used an optimized 48-h fasting protocol that we previously showed maximizes the expression of food anticipatory activity on the second day of food deprivation (after ~40 h of fasting)^18^. We hypothesize that mice must be sufficiently hungry to express food anticipatory activity, which is why anticipatory activity is weak on day 1 of fasting and robust on day 2 of fasting. Food anticipatory wheel-running activity in wild-type, *mPer1* ^*ldc*−/−^, *mPer2* ^*ldc*−/−^, and *mPer3* ^−/−^ mice was robust on the second day of fasting. Our results in *mPer1* ^*ldc*−/−^ mice are consistent with a previous study that showed a different strain of *Per1* ^−/−^ mice also had food anticipatory activity during fasting^10^.

Likewise, some *mPer2* ^*Brdm1*−/−^ mice and their wild-type littermates had food anticipatory activity on the second day of fasting. However, there was individual variability among mice. In addition, a greater proportion of wild-type mice expressed food anticipatory activity on the second day of fasting compared to *mPer2* ^*Brdm1*−/−^ mice. We propose that an optimized fasting protocol may be required for the expression of food anticipatory activity in mutant mice. A previous study did not detect food anticipatory activity during 36-h fasting in *mPer2* ^*Brdm1*−/−^ mice^10^. This could be due to the length of fasting (36-h fasting in the previous study vs. 48-h fasting in our study) and phase when food was removed (24 h before predicted food anticipatory activity in the previous study vs. 42 h before predicted food anticipatory activity in our study). Also, fasting was performed in constant darkness in the previous study, so it was impossible to distinguish the SCN-controlled free-running activity from FEO-controlled anticipatory activity. To avoid this complication, fasting should be performed in the light-dark cycle or in SCN-lesioned mice. In sum, using an optimized fasting protocol, our study showed that the FEO in *mPer2* ^*Brdm1*−/−^ mice is functional and keeps time in the absence of the food cue.

Together our data show that food anticipatory is present in single *Period* mutant mice, including *mPer2* ^*Brdm1*−/−^ mice, during restricted feeding and subsequent food deprivation. These data demonstrate that the FEO is functional in *Period* mutant mice. Moreover, our data further support the hypothesis that the FEO uses a non-canonical timekeeping mechanism and that *Period2* is not critical for expression of food anticipatory activity.

## Methods

### Animals

*Period1* (*mPer1* ^*ldc*−/−^), *Period2* (*mPer2* ^*ldc*−/−^), and *Period3* (*mPer3* ^−/−^) mutant mice^14^ were obtained from Dr. David Weaver on a 129/sv background and backcrossed to Jackson Laboratory C57Bl/6 J mice for 10 to 11 generations. Experimental mice and wild-type controls were generated from heterozygote breeding pairs for each genotype. Mice were born and raised in 12 L:12D at Vanderbilt University and fed chow (LabDiet 5001) *ad libitum*. Genotype was determined by PCR amplification of tail DNA as previously described^14,19^. Male and female mice, aged 6 to 13 weeks at the beginning of the experiment, were used. All procedures at Vanderbilt University were approved by the Institutional Animal Care and Use Committee at Vanderbilt University.

*mPer2* ^*Brdm1*−/−^ mice were purchased from Jackson Laboratory (stock number 003819; genetic background: 129S7/SvEvBrd-Hprt backcrossed to C57BL/6Brd-*Tyr* ^*c-Brd*^ strain for at least 5 generations) and crossed with C57BL/6 J (Jackson Laboratory) for 1 generation to generate *mPer2* ^*Brdm1+/−*^ heterozygous mice. These heterozygotes were intercrossed to generate *mPer2* ^*Brdm1*−/−^ and wild-type control mice for experiments. These mice were born and raised in 12 L:12D at the University of Kentucky and fed chow (Teklad 2918) *ad libitum*. *m* *Per2* ^*Brdm1*−/−^ mice were genotyped according to the Jackson Laboratory protocol. Male and female mice, aged 6 to 18 weeks at the beginning of the experiment, were used. All procedures at University of Kentucky were approved by the Institutional Animal Care and Use Committee at University of Kentucky.

All procedures were conducted in accordance with the guidelines of the National Institutes of Health Guide for the Care and Use of Laboratory Animals.

### Activity recording

Mice were single-housed in cages (33 × 17 × 14 cm) with running wheels (diameter: 11 cm) in light-tight boxes in 12 L:12D and fed *ad libitum* chow (LabDiet 5001 at Vanderbilt and Teklad 2918 at University of Kentucky). At Vanderbilt University (*mPer1* ^*ldc*−/−^, *mPer2* ^*ldc*−/−^, and *mPer3* ^−/−^ mice), light sources were white fluorescent bulbs and light intensity was 250-350 lux at the bottom of the cages. At the University of Kentucky (*mPer2* ^*Brdm1+/+*^ and *mPer2* ^*Brdm1*−/−^ mice), light sources were white LEDs and light intensity was 200-300 lux at the bottom of the cages. Wheel-running revolutions were collected every minute using the ClockLab acquisition system (Actimetrics Inc, Wilmette, IL).

### Restricted Feeding

After several days of *ad libitum* chow, food was removed for 24 h beginning at ZT4. Then food availability was gradually reduced. Food was available from ZT4-12 for 2 days, then from ZT4-10 for 2 days, and then from ZT6-10 for 9-10 days. Mice were then fed *ad libitum* for 6 days. To determine if food anticipatory activity persisted in the absence of the food cue, we fasted the mice for 48 h, beginning at ZT12. The timing of fasting is critical as food anticipatory activity becomes more robust as the length of fasting increases (note that food anticipatory activity is more robust on day 2 of fasting compared to day 1 in all genotypes)^18^.

### Analysis

ClockLab Analysis software was used to make double-plotted actograms (10-min bins, normalized format). Mean activity profiles were generated in ClockLab using the following procedure. For each mouse, an activity profile was generated for 3 days of ad libitum feeding (ALI), 9 days of 4-h restricted feeding (RF), and 6 days of subsequent ad libitum feeding (ALII). Since food anticipatory activity becomes more robust with length of fasting, the entire 48-h of fasting was plotted. Then, the mean activity profiles of all mice of each genotype were plotted. The SEM shown in the activity profiles represents the variability among the mice in the group.

Food anticipatory activity during restricted feeding was defined as the 4 h before and 4 h during food availability (e.g. wheel revolutions between ZT2-10 will be summed for restricted feeding beginning at ZT6). Similarly, food anticipatory activity during fasting was defined as the 4 h before and 4 h during the time when food was previously available. Food anticipatory activity during fasting was quantified separately for the first and second days of fasting. The food anticipatory activity ratio was calculated by dividing the number of wheel revolutions 4 h before and 4 h during food availability by the total number of daily wheel revolutions.

### Data Availability

All data generated or analyzed during this study are included in this published article (and its Supplementary Information files).

## Electronic supplementary material

Supplemental Information
