# Supplementary material for: mPeriod2Brdm1 and other single Period mutant mice have normal food anticipatory activity
Source: Sci Rep. 2017 Nov 14;7:15510. doi: 10.1038/s41598-017-15332-6 (PMC5686205; doi:10.1038/s41598-017-15332-6)
Supplement: Supplementary file 1 — Supplemental Information [file 41598_2017_15332_MOESM1_ESM.pdf]

Supplementary Information

***mPeriod2^{Brdm1}* and other single *Period* mutant mice have normal food anticipatory activity**

Julie S. Pendergast, Robert Wendroth, Rio C. Stenner, Charles D. Keil, and Shin Yamazaki

Supplemental Figures

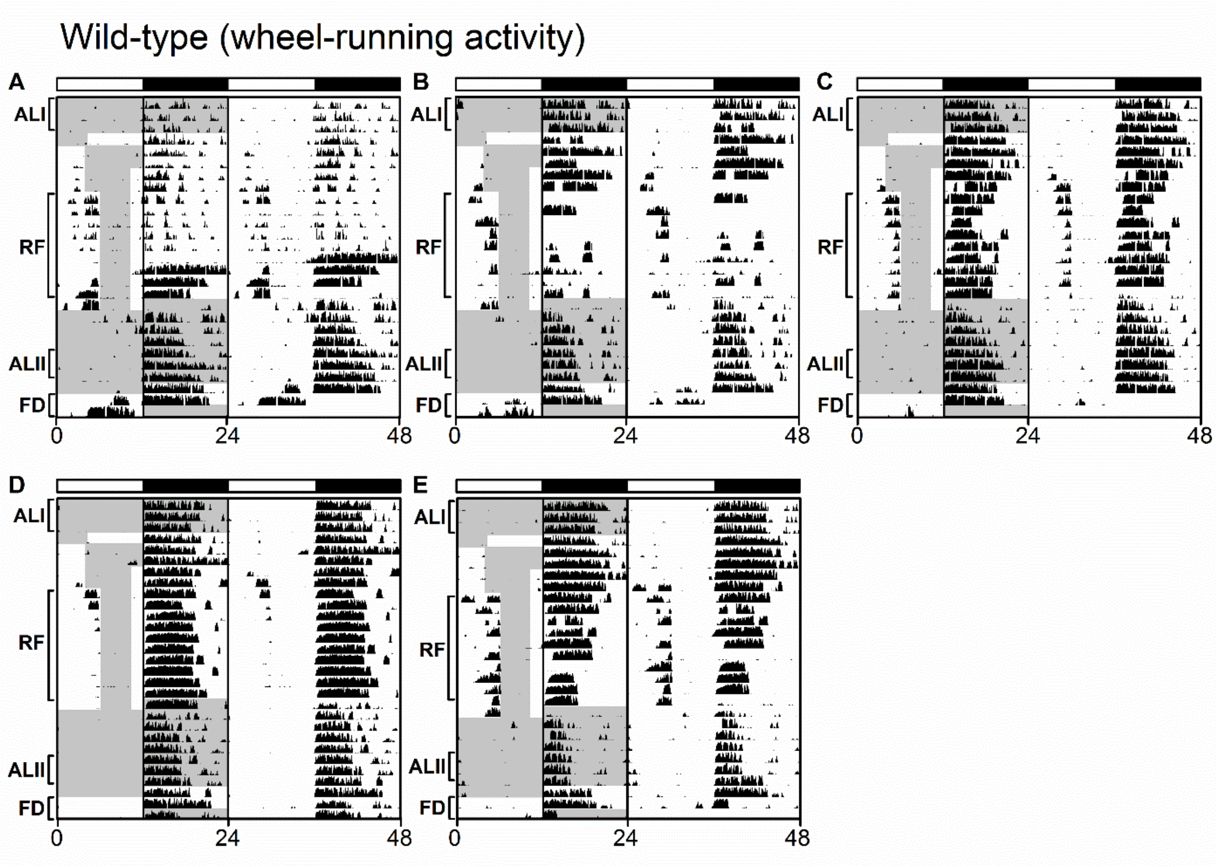

Figure S1. Food anticipatory activity in wild-type (*Per$^{ldc+/+}$*) mice. Double-plotted actograms of wheel-running activity (10-min bins) of wild-type mice (53±1 days old; A-D: males) maintained in 12L:12D (white and black bars above actograms and the dark phase is outlined with a black box on the left half of each actogram). The time when food was available is shown by gray shading on the left half of each actogram. Mice were fed *ad libitum* for 3 days (ALI), then fed 8h/d for 2 days, 6h/day for 2 days, and then 4h/day for 9-10 days (RF). On the 10-11th day of restricted feeding, food was left in the cage and mice ate *ad libitum* for 6 days (ALII). Mice were then fasted for 48h (FD). The data shown in (C) are also shown in Fig. 1A. x-axis: time in hours; y-axis: days.

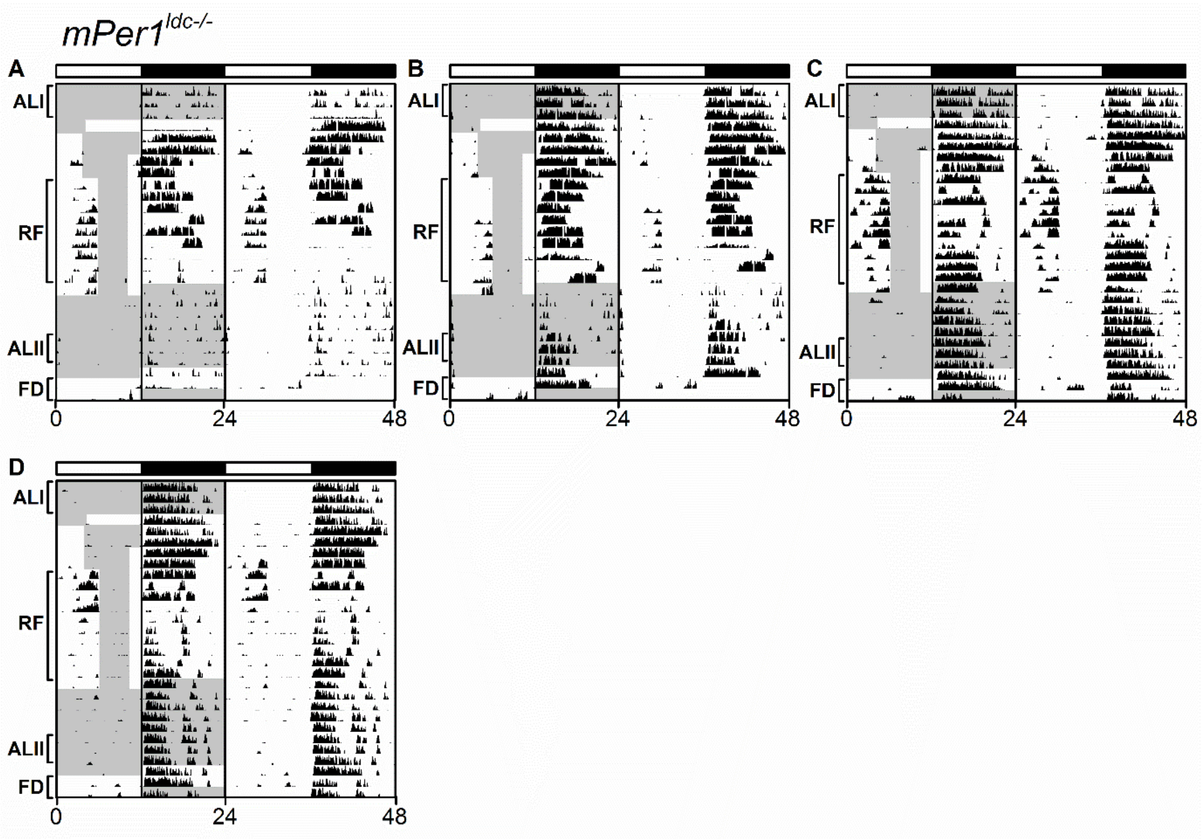

Figure S2. Food anticipatory activity in *mPer1*$^{ldc-/-}$ mice. Double-plotted actograms of wheel-running activity (10-min bins) of *mPer1*$^{ldc-/-}$ mice (56±5 days old; A-D: males) maintained in 12L:12D (white and black bars above actograms and the dark phase is outlined with a black box on the left half of each actogram). The time when food was available is shown by gray shading on the left half of each actogram. Mice were fed *ad libitum* for 3 days (ALI), then fed 8h/d for 2 days, 6h/day for 2 days, and then 4h/day for 9 days (RF). On the 10th day of restricted feeding, food was left in the cage and mice ate *ad libitum* for 6 days (ALII). Mice were then fasted for 48h (FD). x-axis: time in hours; y-axis: days.

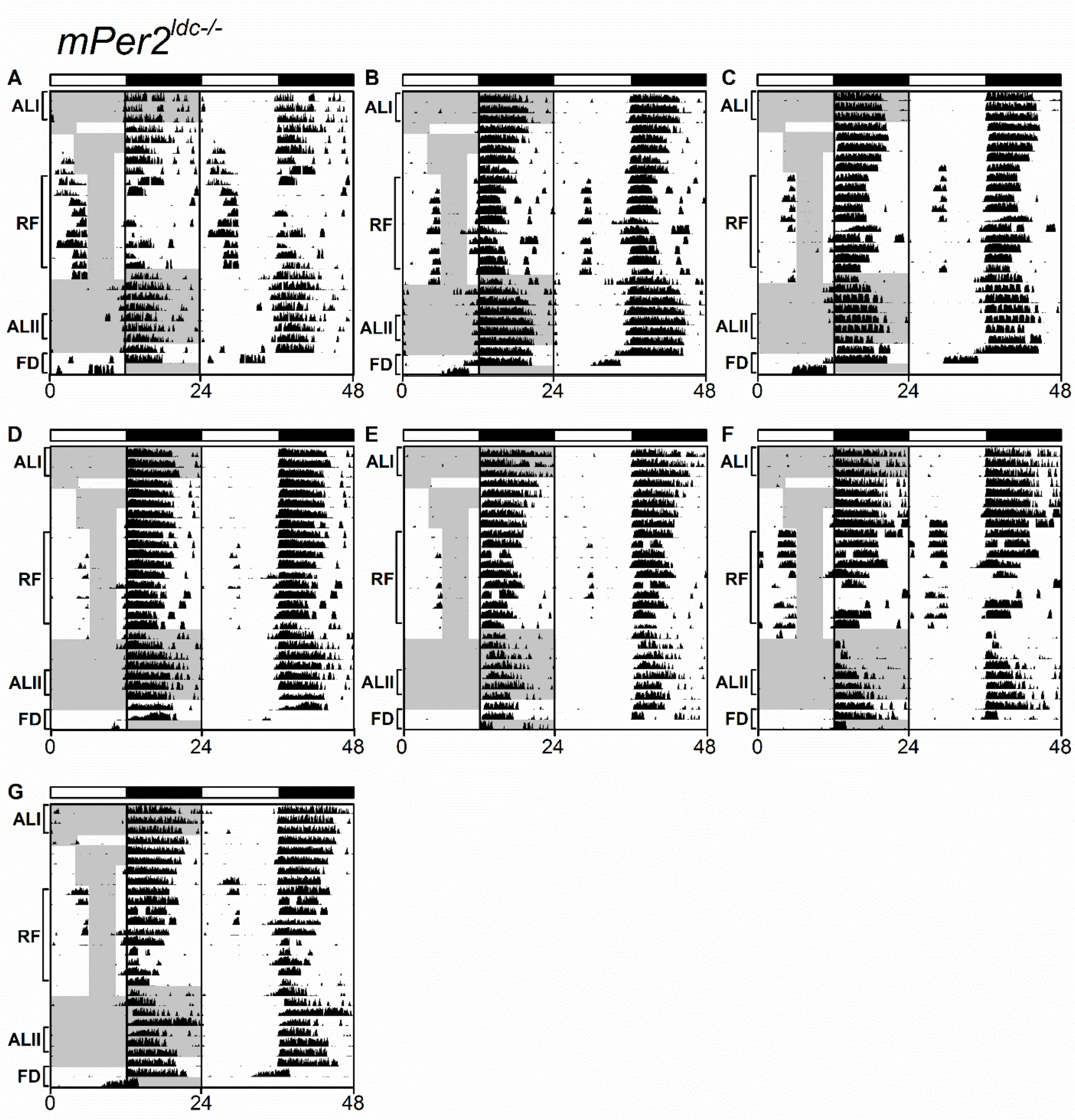

Figure S3. Food anticipatory activity in *mPer2*$^{ldc\text{-}/\text{-}}$ mice. Double-plotted actograms of wheel-running activity (10-min bins) of *mPer2*$^{ldc\text{-}/\text{-}}$ mice (53±2 days old; A: male) maintained in 12L:12D (white and black bars above actograms and the dark phase is outlined with a black box on the left half of each actogram). The time when food was available is shown by gray shading on the left half of each actogram. Mice were fed *ad libitum* for 3 days (ALI), then fed 8h/d for 2 days, 6h/day for 2 days, and then 4h/day for 9-10 days (RF). On the 10-11th day of restricted feeding, food was left in the cage and mice ate *ad libitum* for 6 days (ALII). Mice were then fasted for 48h (FD). x-axis: time in hours; y-axis: days.

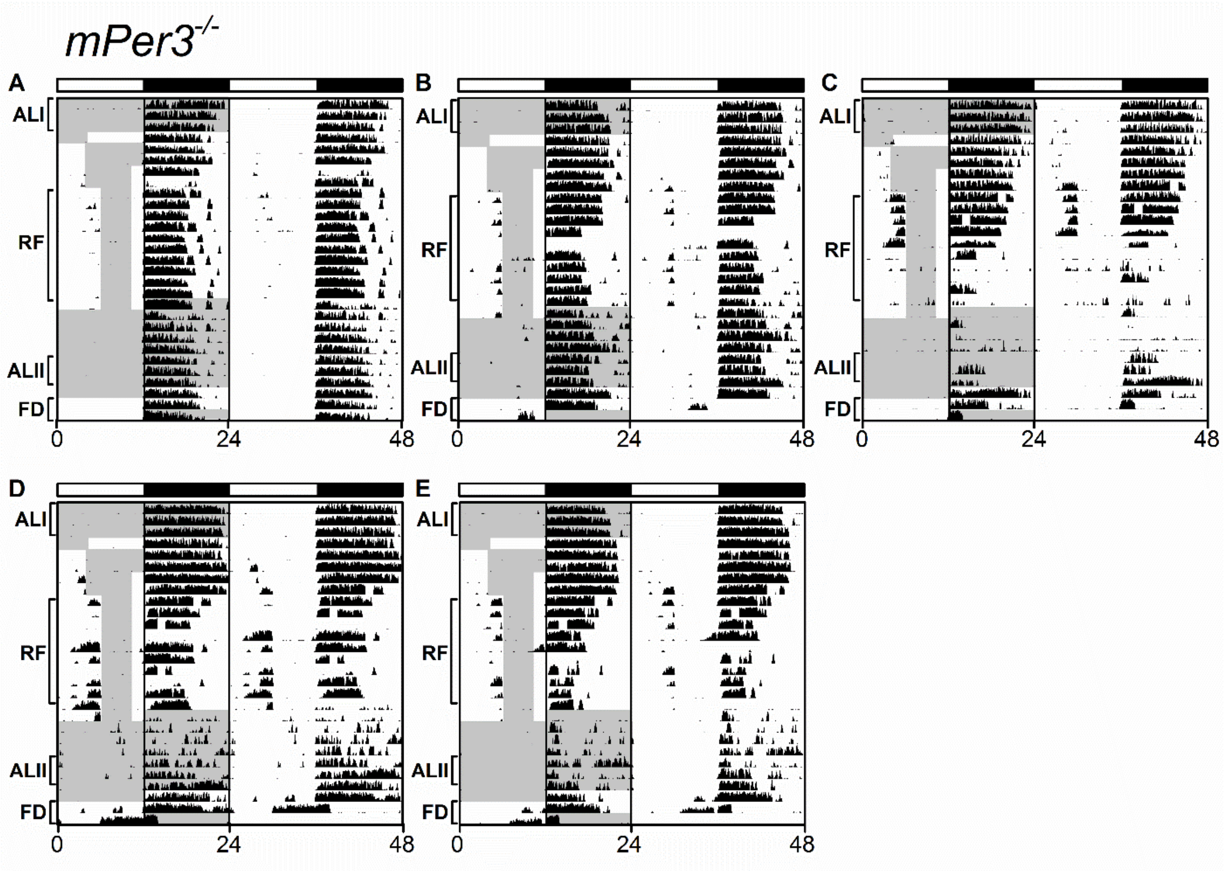

Figure S4. Food anticipatory activity in *mPer3*$^{-/-}$ mice. Double-plotted actograms of wheel-running activity (10-min bins) of *mPer3*$^{-/-}$ mice (58±4 days old; A: male) maintained in 12L:12D (white and black bars above actograms and the dark phase is outlined with a black box on the left half of each actogram). The time when food was available is shown by gray shading on the left half of each actogram. Mice were fed *ad libitum* for 3 days (ALI), then fed 8h/d for 2 days, 6h/day for 2 days, and then 4h/day for 10 days (RF). On the 11th day of restricted feeding, food was left in the cage and mice ate *ad libitum* for 5 days (ALII). Mice were then fasted for 48h (FD). The data shown in (D) are also shown in Fig. 1D. A-Dx-axis: time in hours; y-axis: days.

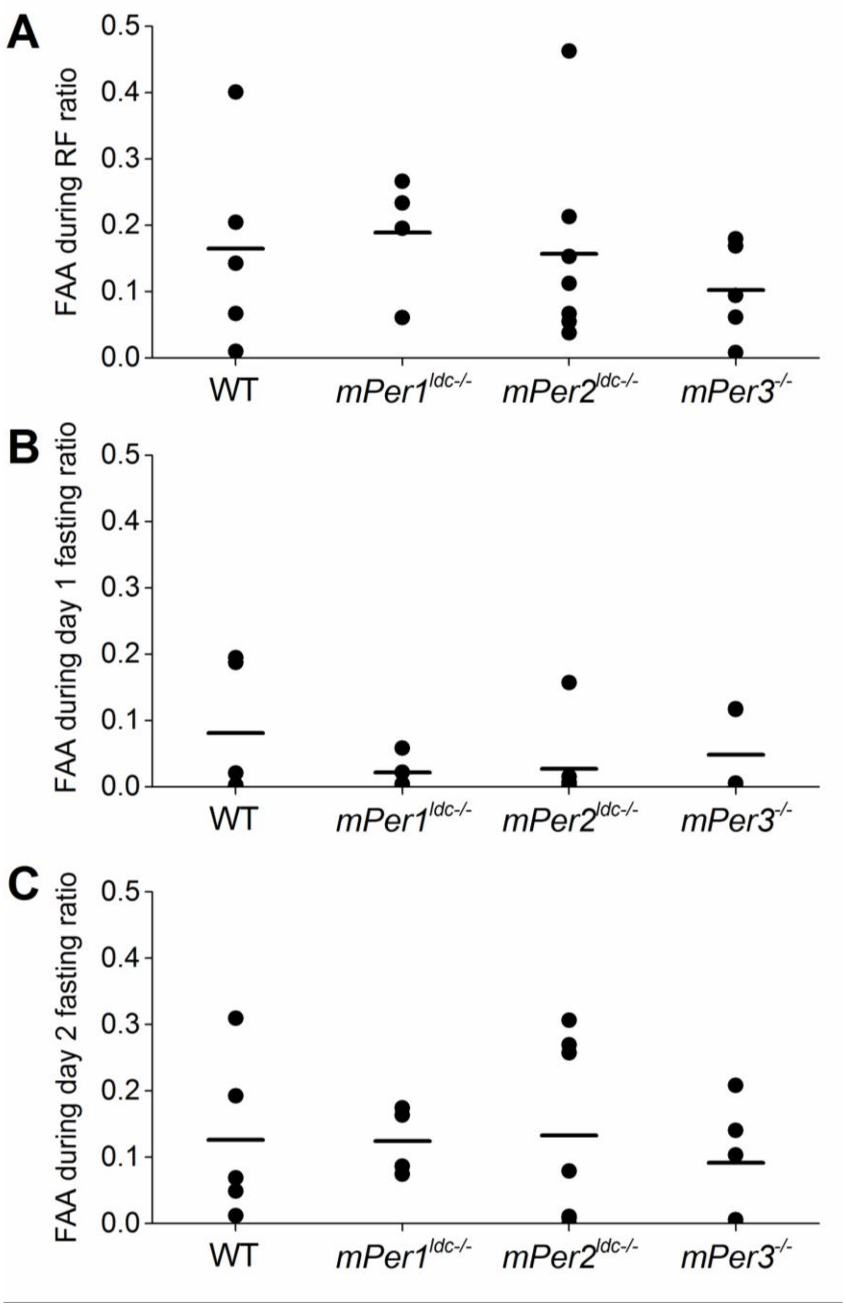

Figure S5. Food anticipatory activity ratio from individual wild-type and *Period* mutant *(ldc strain*) mice. a. Food anticipatory activity (FAA) ratio during 9 days of restricted feeding (RF) of wild-type, *mPer1*$^{ldc-/-}$, *mPer2*$^{ldc-/-}$, and *mPer3*$^{-/-}$ mice was determined by totaling the number of wheel revolutions per minute from 4 hours before feeding time to the end of feeding time (total of 8 hours) and dividing it by the number of daily wheel revolutions. FAA during fasting ratio was defined as the total number of wheel revolutions per minute from 4 hours before feeding time to the end of previous feeding time (total of 8 hours) divided by the number of daily wheel revolutions. Wheel-running FAA for each mouse was determined separately for the first (Day 1; b) or second (Day 2; c) day of fasting. Each black circle is data from one mouse. The mean of each group is a horizontal line.

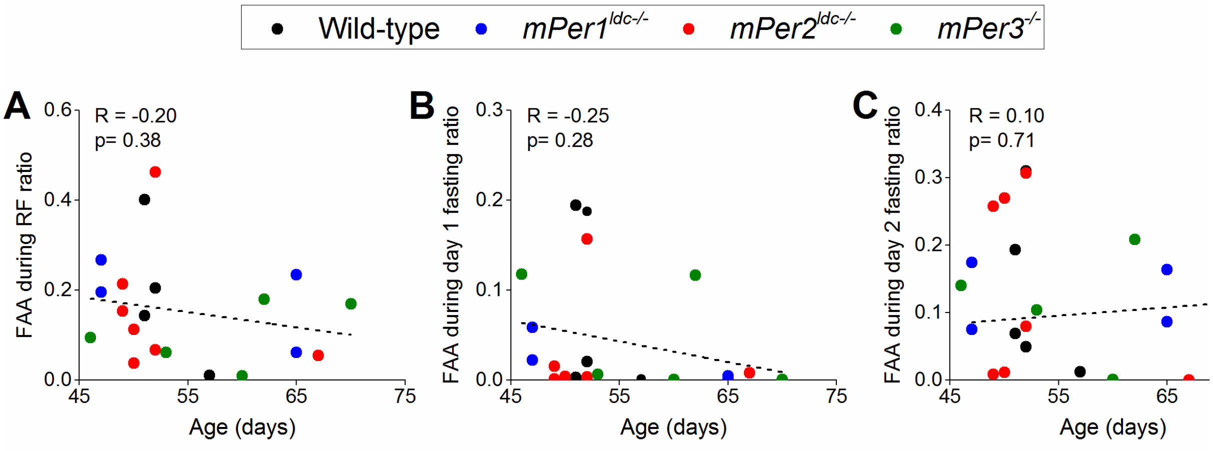

Figure S6. The robustness of FAA does not vary with age in wild-type and *Period* mutant *(ldc strain*) mice. The FAA ratio during restricted feeding (A), day 1 fasting (B), and day 2 fasting (C) of individual wild-type (black), *mPer1*$^{ldc-/-}$ (blue), *mPer2*$^{ldc-/-}$ (red), and *mPer3*$^{-/-}$ (green) plotted relative to their ages.

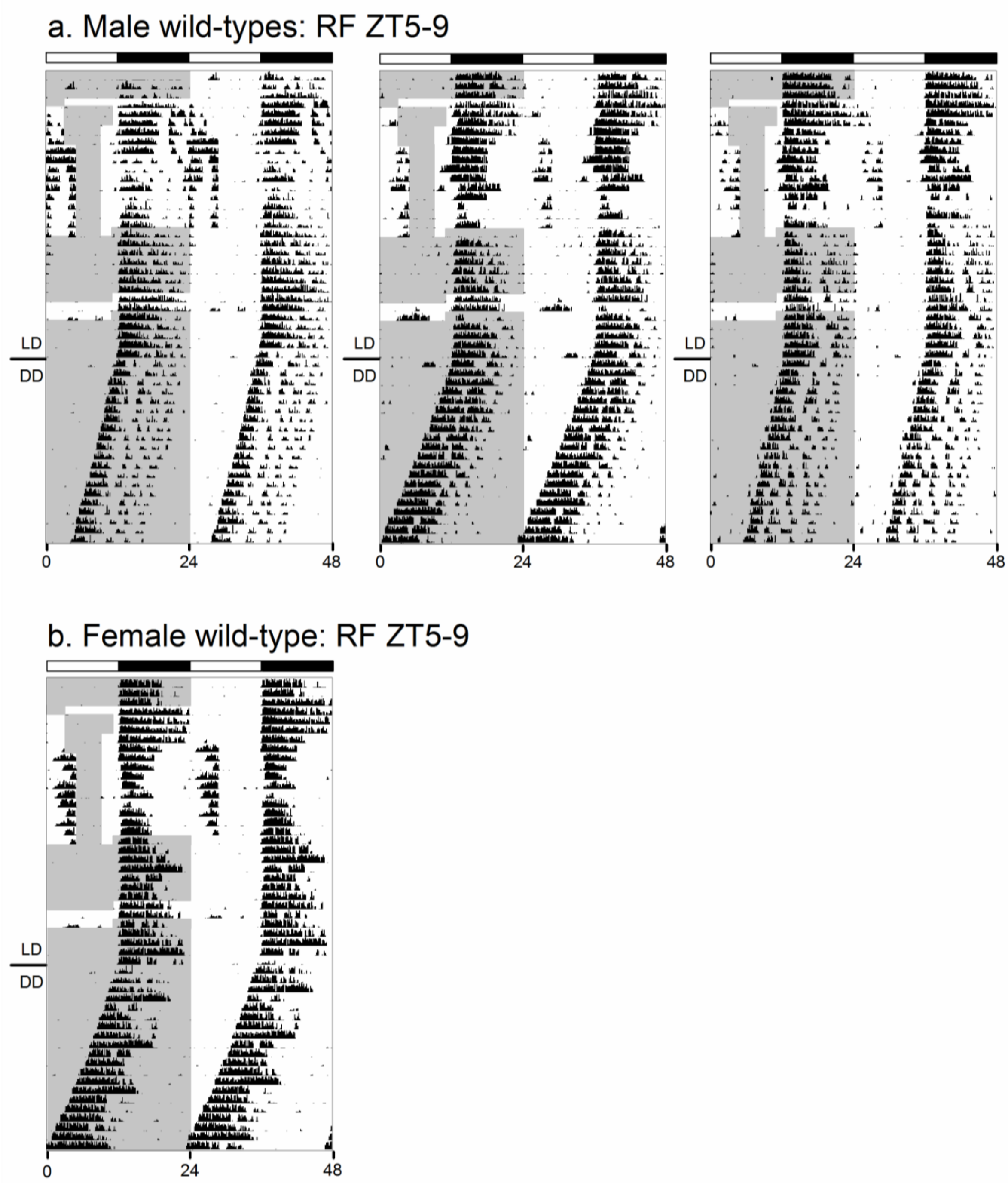

Figure S7. Food anticipatory activity in wild-type (Brdm strain) mice: restricted feeding ZT5 to 9. Double-plotted actograms of wheel-running activity (10-min bins) of male (a) and female (b) wild-type (*Per2*$^{Brdm1+/+}$) mice maintained in 12L:12D (LD; white and black bars above actograms). The time when food was available is shown by gray shading on the left half of each actogram. Mice were fed *ad libitum* for 3 days , then fed 8h/d for 2 days, 6h/day for 2 days, and then 4h/day for 10 days. On the 10th day of restricted feeding, food was put in the cage at ZT11 and mice ate *ad libitum* for 6.5 days. Mice were then fasted for 48h. Then mice were then fed *ad libitum* and released into constant darkness (indicated by DD). x-axis: time in hours; y-axis: days.

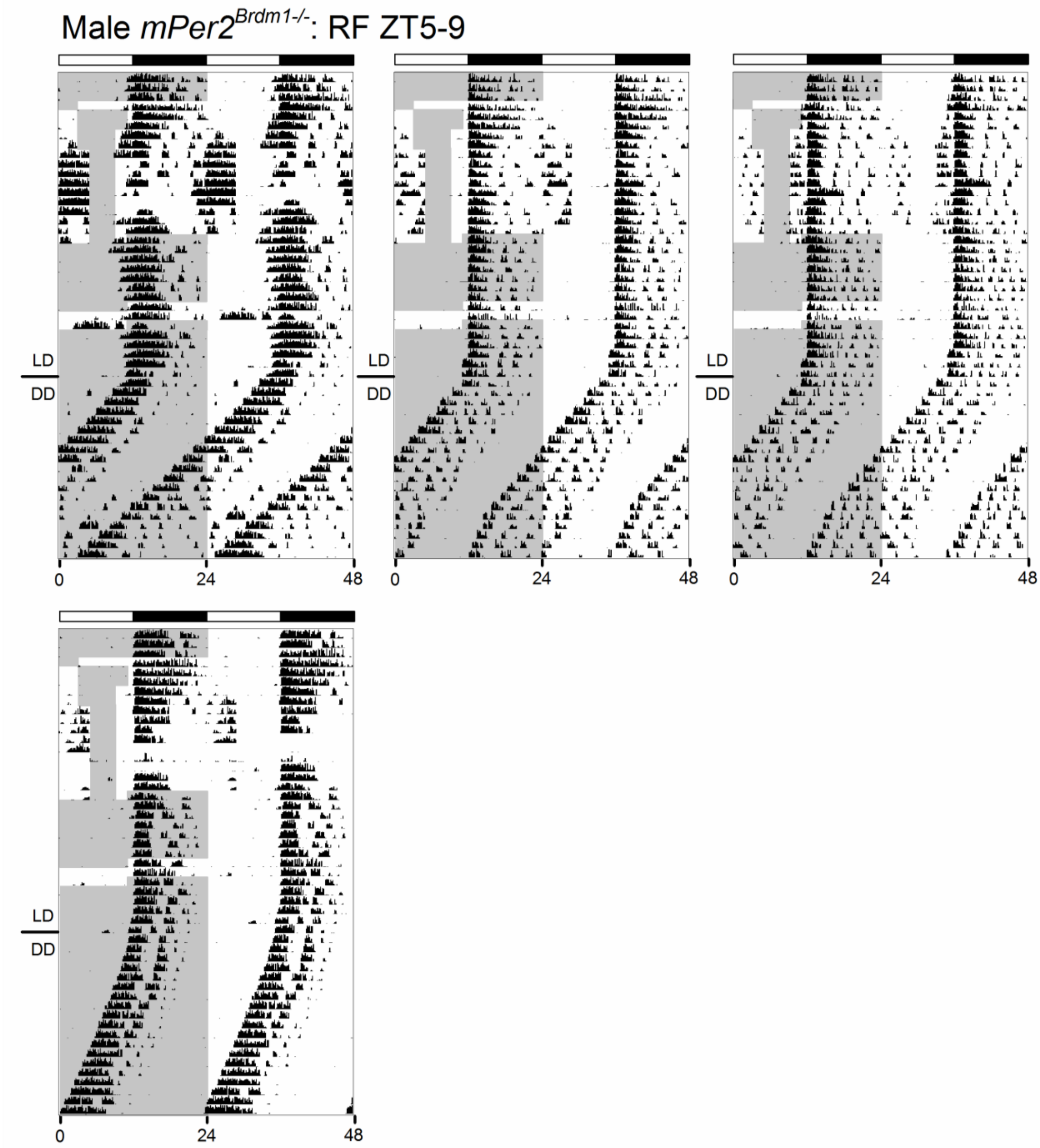

Figure S8. Food anticipatory activity in male *Per2*$^{Brdm1-/-}$ mice: restricted feeding ZT5 to 9. Double-plotted actograms of wheel-running activity (10-min bins) of male *Per2*$^{Brdm1-/-}$ mice maintained in 12L:12D (LD; white and black bars above actograms). The time when food was available is shown by gray shading on the left half of each actogram. Mice were fed *ad libitum* for 3 days , then fed 8h/d for 2 days, 6h/day for 2 days, and then 4h/day for 10 days. On the 10th day of restricted feeding, food was put in the cage at ZT11 and mice ate *ad libitum* for 6.5 days. Mice were then fasted for 48h. Then mice were then fed *ad libitum* and released into constant darkness (indicated by DD). x-axis: time in hours; y-axis: days.

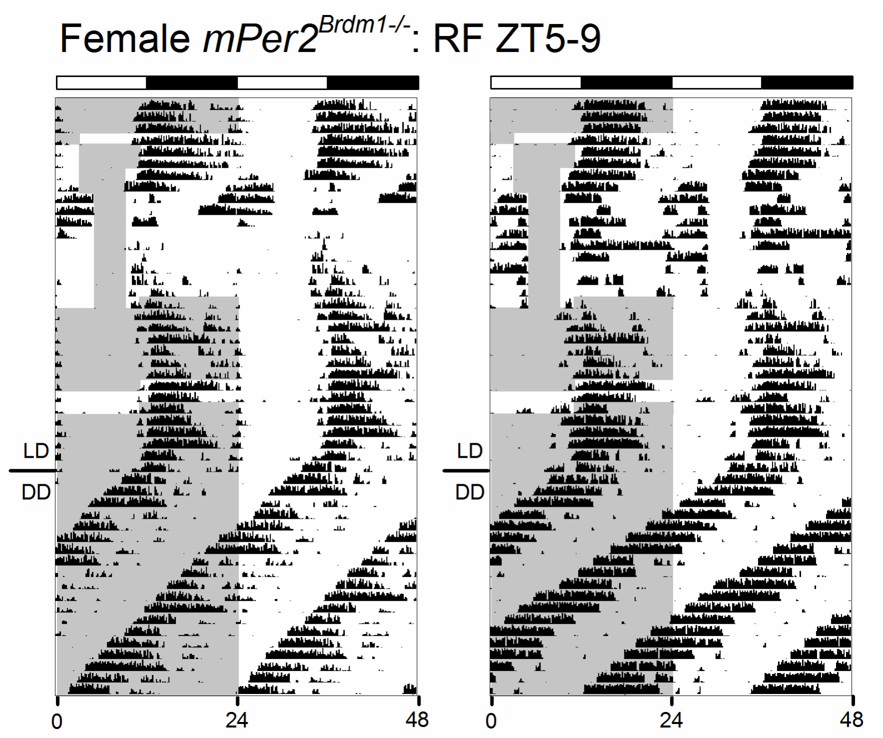

Figure S9. Food anticipatory activity in female *Per2*$^{Brdm1\text{-}/\text{-}}$ mice: restricted feeding ZT5 to 9. Double-plotted actograms of wheel-running activity (10-min bins) of male *Per2*$^{Brdm1\text{-}/\text{-}}$ mice maintained in 12L:12D (LD; white and black bars above actograms). The time when food was available is shown by gray shading on the left half of each actogram. Mice were fed *ad libitum* for 3 days , then fed 8h/d for 2 days, 6h/day for 2 days, and then 4h/day for 10 days. On the 10th day of restricted feeding, food was put in the cage at ZT11 and mice ate *ad libitum* for 6.5 days. Mice were then fasted for 48h. Then mice were then fed *ad libitum* and released into constant darkness (indicated by DD). x-axis: time in hours; y-axis: days.

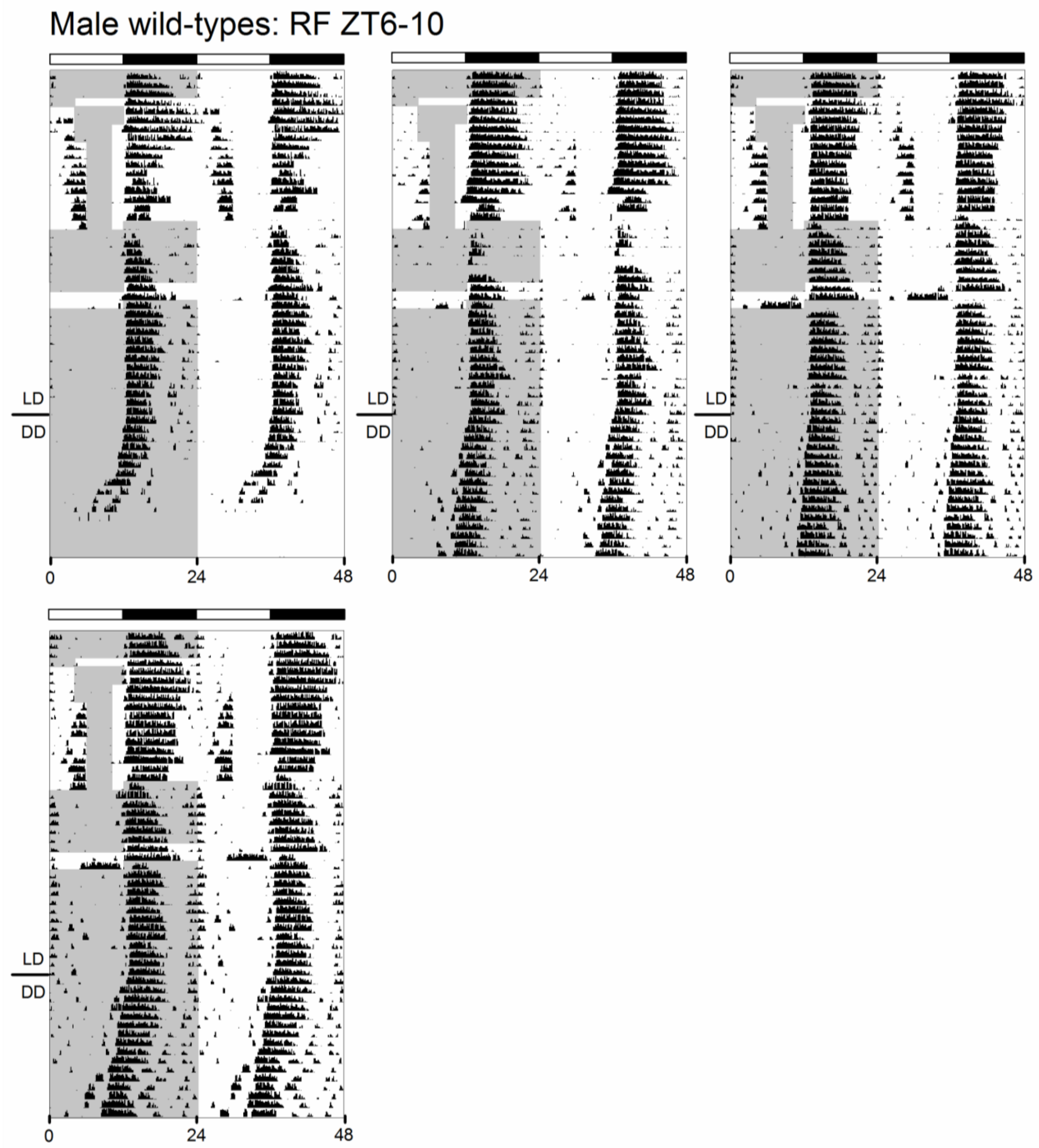

Figure S10. Food anticipatory activity in male wild-type (Brdm strain) mice: restricted feeding ZT6 to 10. Double-plotted actograms of wheel-running activity (10-min bins) of male wild-type (*Per2*$^{Brdm1+/+}$) mice maintained in 12L:12D (LD; white and black bars above actograms). The time when food was available is shown by gray shading on the left half of each actogram. Mice were fed *ad libitum* for 3 days , then fed 8h/d for 2 days, 6h/day for 2 days, and then 4h/day for 10 days. On the 10th day of restricted feeding, food was put in the cage at ZT11 and mice ate *ad libitum* for 6.5 days. Mice were then fasted for 48h. Then mice were then fed *ad libitum* and released into constant darkness (indicated by DD). x-axis: time in hours; y-axis: days.

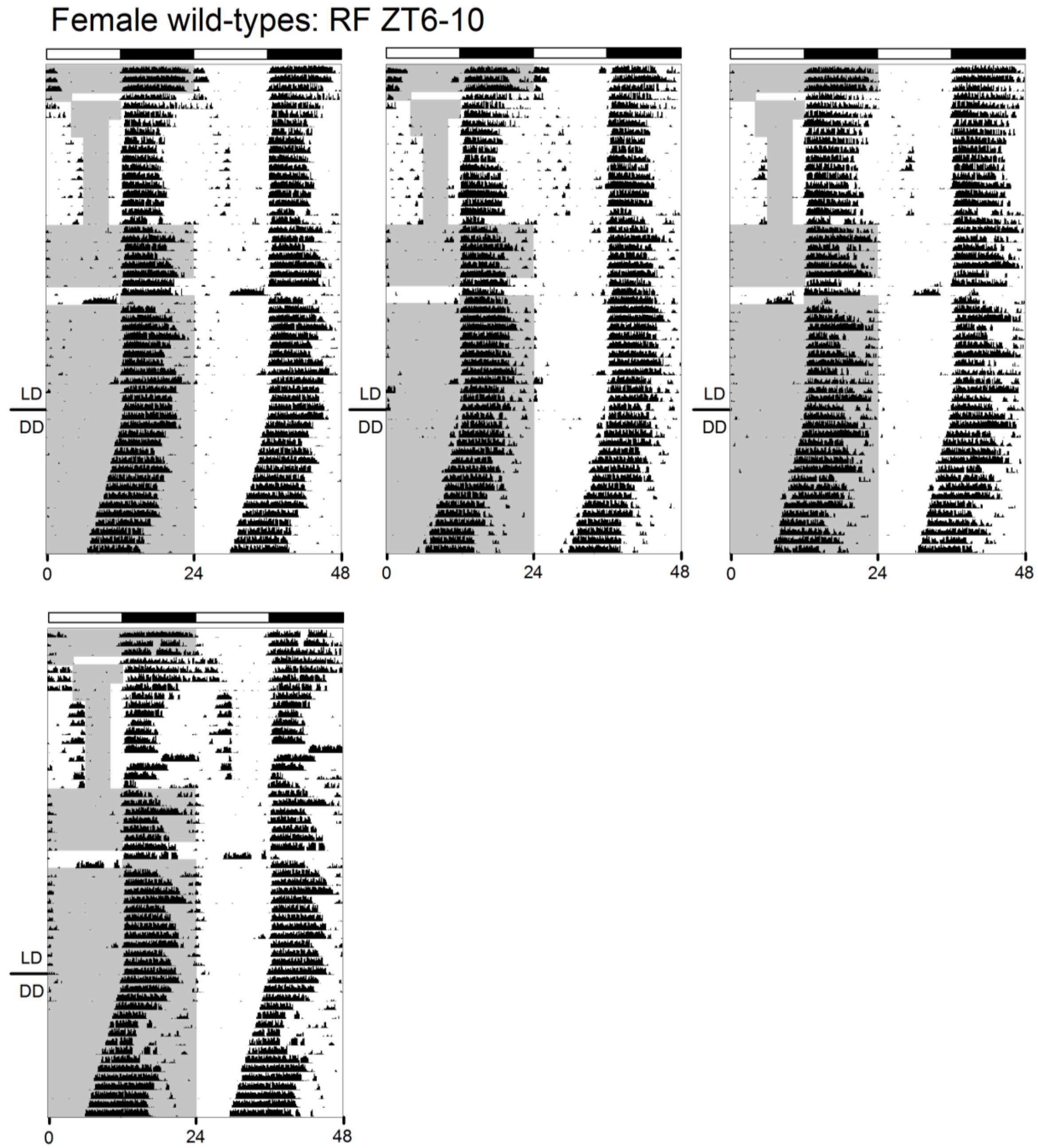

Figure S11. Food anticipatory activity in female wild-type (Brdm strain) mice: restricted feeding ZT6 to 10. Double-plotted actograms of wheel-running activity (10-min bins) of female wild-type (*Per2*$^{Brdm1+/+}$) mice maintained in 12L:12D (LD; white and black bars above actograms). The time when food was available is shown by gray shading on the left half of each actogram. Mice were fed *ad libitum* for 3 days, then fed 8h/d for 2 days, 6h/day for 2 days, and then 4h/day for 10 days. On the 10th day of restricted feeding, food was put in the cage at ZT11 and mice ate *ad libitum* for 6.5 days. Mice were then fasted for 48h. Then mice were then fed *ad libitum* and released into constant darkness (indicated by DD). x-axis: time in hours; y-axis: days.

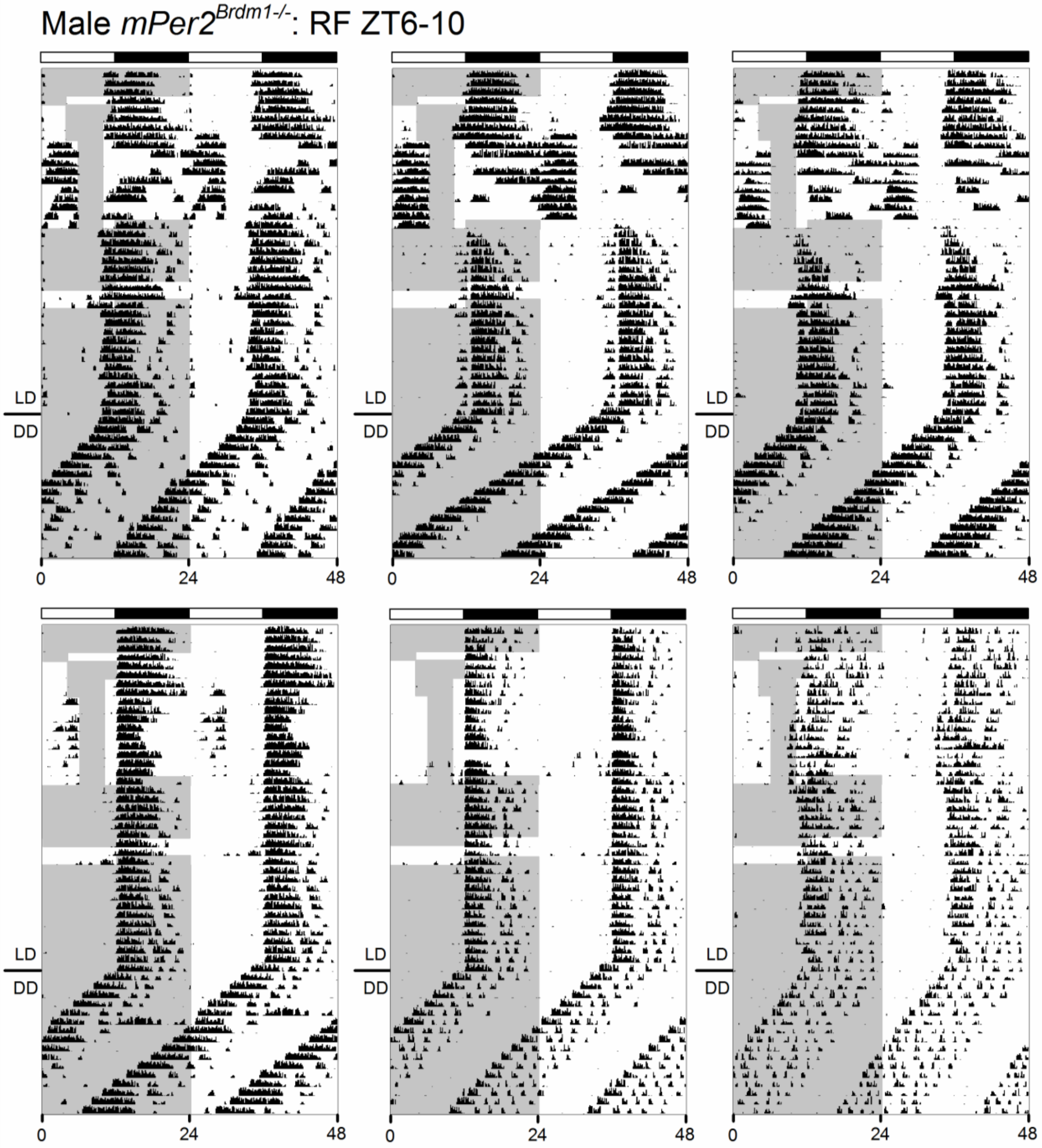

Figure S12. Food anticipatory activity in male *Per2*$^{Brdm1-/-}$ mice: restricted feeding ZT6 to 10. Double-plotted actograms of wheel-running activity (10-min bins) of male *Per2*$^{Brdm1-/-}$ mice maintained in 12L:12D (LD; white and black bars above actograms). The time when food was available is shown by gray shading on the left half of each actogram. Mice were fed *ad libitum* for 3 days , then fed 8h/d for 2 days, 6h/day for 2 days, and then 4h/day for 10 days. On the 10th day of restricted feeding, food was put in the cage at ZT11 and mice ate *ad libitum* for 6.5 days. Mice were then fasted for 48h. Then mice were then fed *ad libitum* and released into constant darkness (indicated by DD). x-axis: time in hours; y-axis: days.

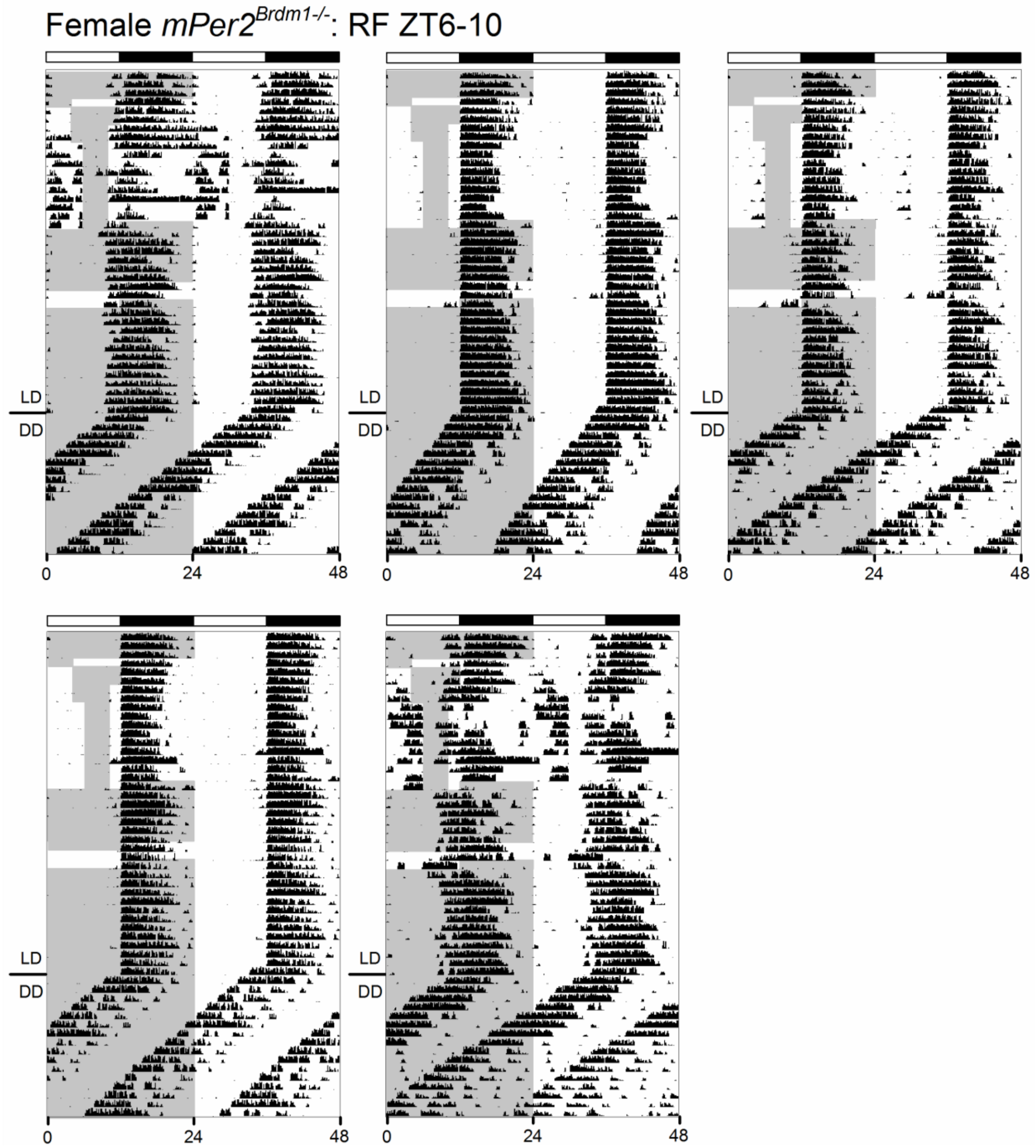

Figure S13. Food anticipatory activity in female *Per2*$^{Brdm1-/-}$ mice: restricted feeding ZT6 to 10. Double-plotted actograms of wheel-running activity (10-min bins) of female *Per2*$^{Brdm1-/-}$ mice maintained in 12L:12D (LD; white and black bars above actograms). The time when food was available is shown by gray shading on the left half of each actogram. Mice were fed *ad libitum* for 3 days , then fed 8h/d for 2 days, 6h/day for 2 days, and then 4h/day for 10 days. On the 10th day of restricted feeding, food was put in the cage at ZT11 and mice ate *ad libitum* for 6.5 days. Mice were then fasted for 48h. Then mice were then fed *ad libitum* and released into constant darkness (indicated by DD). x-axis: time in hours; y-axis: days.

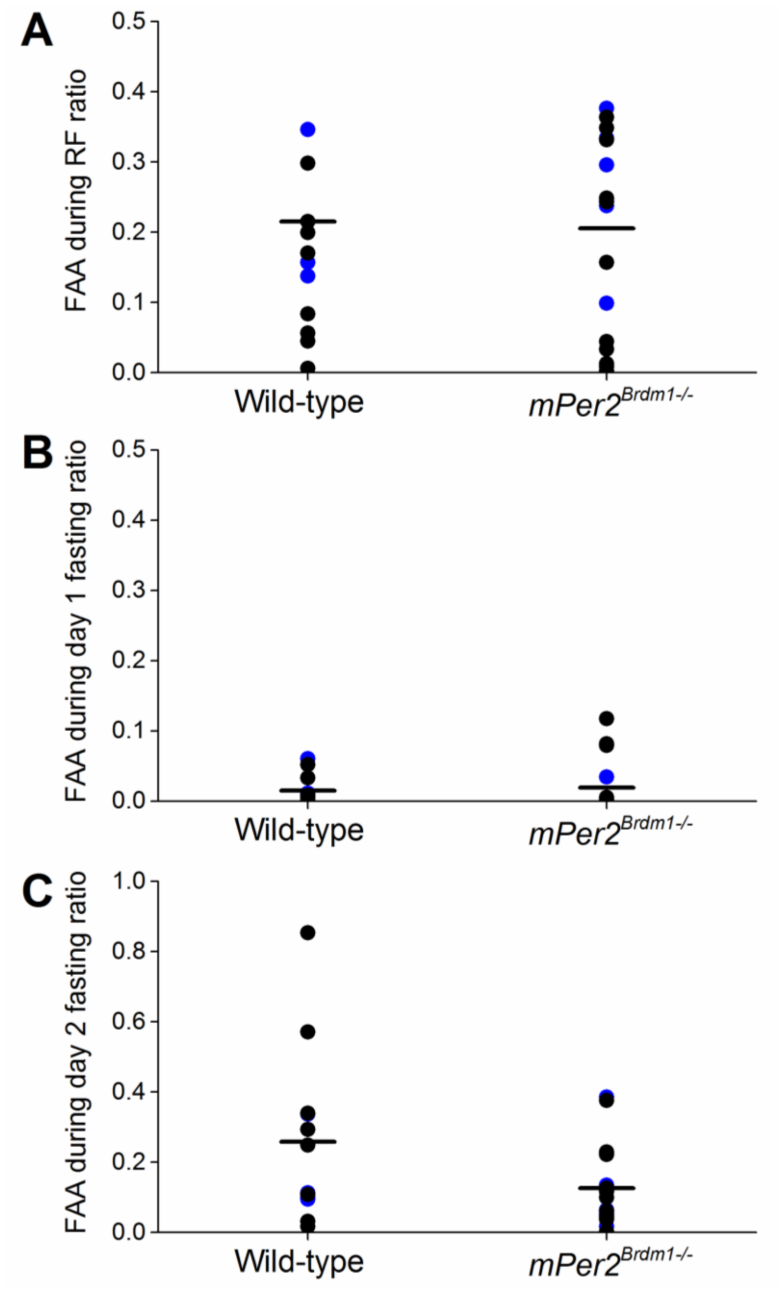

Figure S14. Food anticipatory activity ratio from individual wild-type and *Period* mutant *(Brdm strain*) mice. a. Food anticipatory activity (FAA) ratio during 9 days of restricted feeding (RF) of wild-type and *mPer2*$^{Brdm1-/-}$ mice was determined by totaling the number of wheel revolutions per minute from 4 hours before feeding time to the end of feeding time (total of 8 hours) and dividing it by the number of daily wheel revolutions. FAA during fasting ratio was defined as the total number of wheel revolutions per minute from 4 hours before feeding time to the end of previous feeding time (total of 8 hours) divided by the number of daily wheel revolutions. Wheel-running FAA ratio for each mouse was determined separately for the first (Day 1; b) or second (Day 2; c) day of fasting. Black circles are individual mice fed from ZT6-10. Blue circles are individual mice fed from ZT5-9. The mean of each group is a horizontal line.

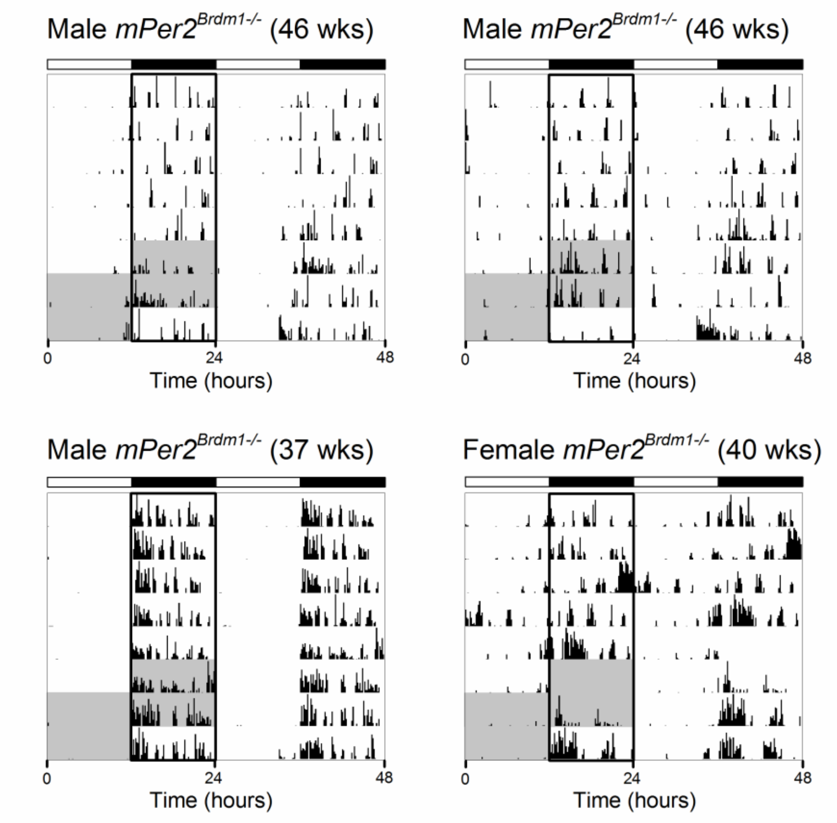

Figure S15. Naïve (no restricted feeding) *mPer2*$^{Brdm1-/-}$ mice do not have elevated daytime activity during fasting. *mPer2*$^{Brdm1-/-}$ mice (sex and age given for each mouse) were single-housed for 1 week with running wheels in 12L:12D and fed *ad libitum*. The mice were then fasted for 48h beginning at ZT12. Gray shading indicates the time of fasting. Note that mice were never exposed to restricted feeding.

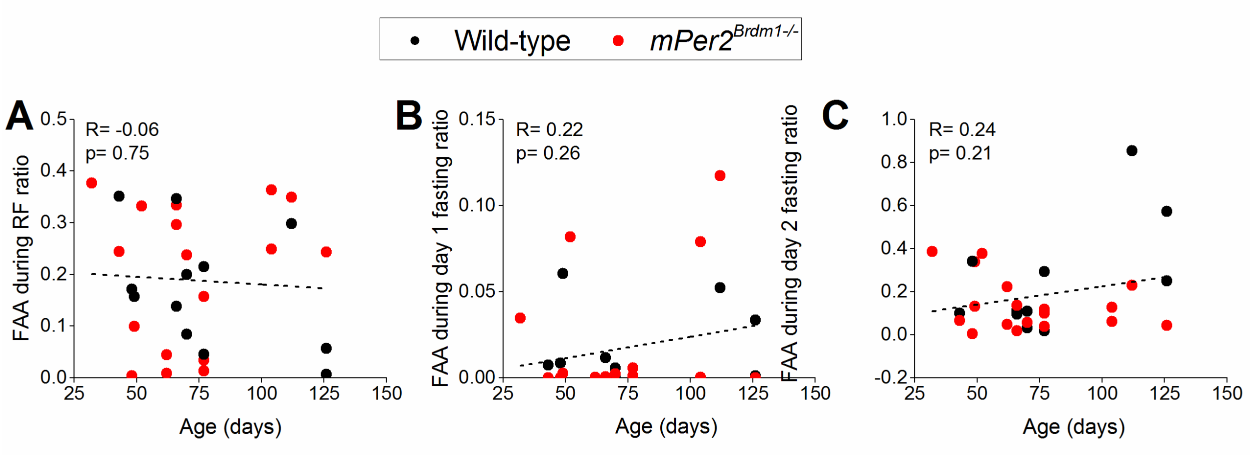

Figure S16. The robustness of FAA does not vary with age in wild-type and *Period* mutant *(Brdm strain*) mice. The FAA ratio during restricted feeding (A), day 1 fasting (B), and day 2 fasting (C) of individual wild-type (black) and *mPer2*$^{Brdm1-/-}$ (red) mice plotted relative to their ages.
